# The Effect of Vitamins on Glaucoma: A Systematic Review and Meta-Analysis

**DOI:** 10.3390/nu10030359

**Published:** 2018-03-16

**Authors:** Wishal D. Ramdas, Jan S. A. G. Schouten, Carroll A. B. Webers

**Affiliations:** 1 Department of Ophthalmology, Maastricht University Medical Center, 6229 HX Maastricht, The Netherlands; j.schouten@mumc.nl (J.S.A.G.S.); c.webers@mumc.nl (C.A.B.W.); 2 Department of Ophthalmology, Erasmus Medical Center, 3000 CA Rotterdam, The Netherlands

**Keywords:** glaucoma, vitamins, nitric oxide, blood levels, dietary intake

## Abstract

Background: The aim of is to determine the association of vitamins with glaucoma by performing a systematic review and meta-analyses. Methods: Studies on the relation of vitamins and glaucoma published up to December 2017 were identified in the PubMed and Embase database. Data on vitamins (method of assessment), glaucoma (type and method of assessment), study characteristics and quality were recorded. In case of multiple studies for one nutrient a meta-analysis was performed. Results: A total of 629 articles were identified of which 36 were included in the systematic review. The meta-analysis included five of them (940 open-angle glaucoma (OAG) cases and 123,697 controls in total) and resulted in an odds ratio [95% confidence interval] (OR [95% CI]) of 0.58 [0.37–0.91] for dietary vitamin A, though heterogeneity was high (I^2^ = 51%). After omitting studies that contributed significantly to the heterogeneity, the pooled OR [95% CI] was 0.45 [0.30–0.68] for dietary vitamin A on OAG (I^2^ = 0%). For vitamin B1, C and E no significant association with OAG was found (OR [95% CI]: 0.84 [0.47–1.51]; 0.68 [0.38–1.22]; 0.95 [0.75–1.19]; respectively). However, after addressing heterogeneity, vitamin C showed a protective effect as well. Especially, foods high in these vitamins (e.g., dark green vegetables) were protective for OAG. Conclusions: Dietary intake of vitamin A and C showed a beneficial association with OAG; however, findings on blood levels of vitamins do not show a clear relation with OAG.

## 1. Introduction

Exposure to nutrients is ubiquitous. Without it, we generally cannot survive and an exposure too high or too low causes health problems. Vitamins are a good example and the ubiquitous exposure to vitamins has led to many epidemiological studies for many diseases. Glaucoma is no exception [1]. The need to study the occurrence of glaucoma in relation to vitamins stems from the observation that glaucoma is the leading cause of irreversible blindness, that almost half of the glaucoma cases are undiagnosed, and that the prevalence is increasing over time [2,3]. This makes it relevant to investigate whether vitamins have a preventive effect on the occurrence of glaucoma i.e., may be used as a preventive measure. Several studies suggested that nutrition might have an effect on the intraocular pressure (IOP; the only treatable risk factor of glaucoma) or glaucoma, mediated by oxidative stress. Oxidative stress occurs when more reactive oxygen species are formed then the anti-oxidative capacity of the cell can handle. This leads to damage of the aqueous humour outflow system of the eye, the trabecular meshwork, resulting in an increase in IOP and eventually loss of retinal ganglion cells [4,5,6]. Moreover, a low ocular (in the aqueous humour) or systemic anti-oxidative capacity is associated with more severe glaucomatous visual field loss [5,7,8]. Although several studies have been conducted on the association of vitamins with glaucoma, it is often noticed that the results are conflicting leaving physicians and patients in doubt about the effect of vitamins on glaucoma.

To address the conflicting results of the association of vitamins with glaucoma, we performed a systematic review and meta-analyses to summarize the evidence. In this review, we assess the quality of the studies, calculate the pooled effect size and explain the heterogeneity between studies. Finally we report the level of evidence of a protective effect of vitamins on the occurrence of glaucoma.

## 2. Methods

The systematic review was performed according to the reporting guidelines implied by the Preferred Reporting Items for Systematic reviews and Meta-Analyses (PRISMA) and Meta-analysis Of Observational Studies in Epidemiology (MOOSE) [9,10].

### 2.1. Search Strategy and Study Eligibility

We conducted a PubMed and Embase database search for articles published prior to 1 December 2017. The search term used in PubMed included (“vitamin (All Fields) AND glaucoma (All Fields)”). The included entries had to have an available abstract, which had to be either in English or German. Furthermore, investigations had to be performed in humans (or human tissue) and not in animals.

Titles and abstracts were scanned to select eligible articles in which the relation between vitamins and glaucoma was studied without any restricting selection criteria. Next, the full text of the remaining studies was used to select additional studies from the reference lists. Studies on the dietary association of nutrition had to use (semiquantitative) food frequency questionnaires, and/or a detailed interview in case of vitamin supplements use, and had to use clear criteria for defining the type of glaucoma for final inclusion if applicable. The most common forms of glaucoma: primary open-angle glaucoma (POAG), primary angle-closure glaucoma (PACG), normal-tension glaucoma (NTG), and pseudoexfoliation (PEX) glaucoma were considered eligible. Other causes of glaucoma were excluded.

### 2.2. Data Extraction and Quality Assessment

The studies were described according to a method described elsewhere [11]. For each study we extracted the names of authors, title, year of publication, study design, sample size, definition of type of glaucoma, effect estimates, exposure assessment method, and confounders. Next, to assess the methodological quality within individual studies the Newcastle-Ottawa Scale (NOS) for assessing the quality of comparative nonrandomized studies was used for the retrieved studies [12]. Studies were classified according to different vitamins: A, B-complex, C, D, and E.

Per nutrient we firstly describe the possible mechanism regarding to its possible influence in glaucoma. Secondly, we provide an overview of studies that investigated the nutrient in question in relation to blood or aqueous humour levels in patients with glaucoma. Thirdly, we provide an overview of the studies that investigated the association of intake of the nutrient in question with glaucoma. Finally, we summarize the evidence from the literature on the association of both blood levels and dietary intake of vitamins, with glaucoma. For this purpose all investigators (WDR, JSAGS, and CABW) independently judged/scored whether a vitamin was not (0), possibly (1), probably (2), or definitely (3) associated with glaucoma. They were blinded for the study that assessed the association of the vitamin with glaucoma. When assessing the evidence for an association, they were given the following data: the number of studies that tested an association, the sample size of the studies, the prevalence of glaucoma in the studies, the quality of the studies, and whether there was a (statistically significant) difference. Next, a final score per nutrient was presented per vitamin.

### 2.3. Data Synthesis and Analysis

Results of retrieved studies are presented as Hazard Ratio (HR) or Odds Ratio (OR) with corresponding 95% confidence interval (CI) if provided. Meta-analyses were performed to calculate the summary effect estimate of the relation between the vitamin and glaucoma.

If for a specific nutrient multiple studies presented their effect size on glaucoma, a meta-analysis using fixed- or random-effects models was performed to calculate the combined effect through the different studies for the association of the specific nutrient with glaucoma. Heterogeneity of the meta-analyses was measured by calculating the I^2^ [13]. In case of homogeneity (or low heterogeneity) the fixed effects model was used. If heterogeneity was substantial (I^2^ > 50%) the random effects model was used. If heterogeneity still remained high (I^2^ > 50%), the study that created the heterogeneity was excluded. This was done by sequentially omitting one study and reanalyzing the pooled estimate for the remaining studies [14]. The study that caused the heterogeneity was compared in detail to the other studies to find differences that could explain the heterogeneity. 

A few articles used a different measure of effect size (HR). It has been suggested that these may not be pooled in a meta-analysis with OR’s [15] (Cochrane website). However, in cohort studies with a low disease prevalence or incidence (<20%) in the study population the OR is close to the risk ratio, which can be combined with HR’s in a pooled meta-analysis [16] (Cochrane website).

Statistical analyses were performed using RevMan version 5.3 for Mac (Copenhagen: The Nordic Cochrane Centre, The Cochrane Collaboration, 2014) and R statistical package version 2.11.1 for Mac (www.r-project.org).

## 3. Results

**Figure 1:**
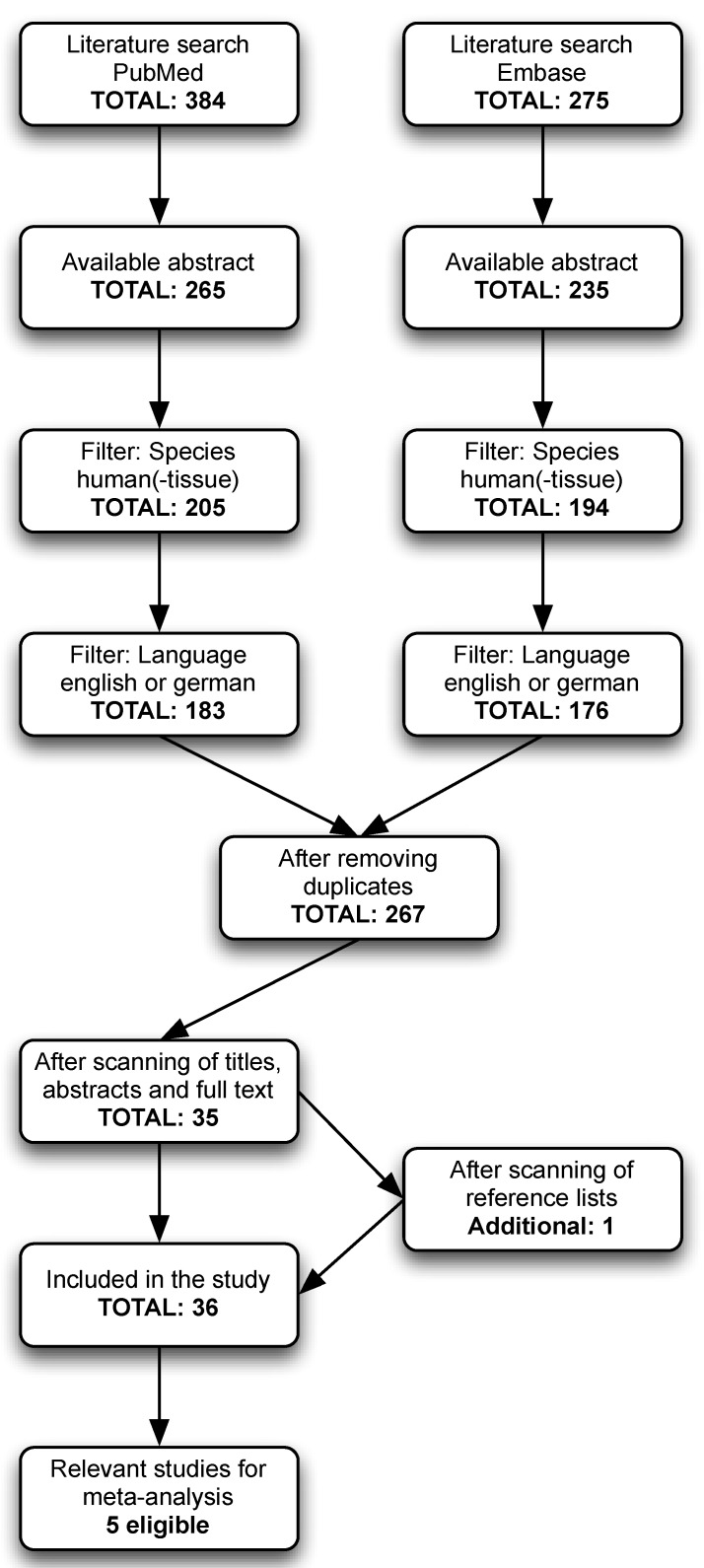
Flow diagram (according to PRISMA) showing the selection process for inclusion of studies from PubMed and Embase.

The literature search yielded a total of 384 articles from PubMed and 275 from the Embase database. After exclusion of those without an available abstract, 265 and 235 articles remained, respectively. Screening of the reference lists of included articles yielded one additional study. Finally, a total of 36 articles were included of which five were eligible for a meta-analysis (Figure 1; Supplementary Table S1). Regarding the quality rating of each of the included studies, the mean total quality score for all studies was 6.8 (interquartile range: 6 to 8), on a scale from 0 to 9 (Supplementary Table S2). Table 1 presents an overview of the investigated vitamins retrieved from the search results: vitamins A, B1, B2, B3, B6, B9, B12, C, D, and E, with their corresponding properties and food sources. Several studies assessed the association of blood levels of vitamins with several forms of glaucoma (Supplementary Table S3).

Vitamin A is known for its function in the retina. Five studies reported on the association of blood levels of vitamin A and glaucoma. One study reported higher vitamin A levels in patients with POAG compared to NTG [17] and one also to controls [18]. Three other studies could not find a significant difference [19,20,21]. In contrast, regarding to dietary intake of retinol equivalents two large studies reported a protective effect on OAG [1,22]. A borderline significant association was found in the Study on osteoporotic fractures in women [23]. However, the Nurses’ Health Study and Health Professionals Follow-up Study and NHANES did not find a significant association between vitamin A and self-reported POAG [21,24].

Vitamin B-complex-investigated vitamins are B1, B2, B3, B6, B9, and B12. Nineteen studies reported on the association of blood levels of vitamin B-complex and glaucoma. Of these, there was one on vitamin B1, none on vitamin B2 or B3, three on B6, eight on B9, and seven on B12. Patients with OAG have significant lower serum levels of vitamin B1 and B12 compared to controls in two studies [25,26]. Others reported no significant differences in plasma levels of vitamin B6 or serum levels of B9 or B12 in patients with OAG compared to controls [17,20,27,28,29,30]. However, lower vitamin B9 and B12 are strongly related to higher homocysteine. Homocysteine generates oxidative stress and induces apoptosis in retinal ganglion cells. Increased levels of homocysteine in aqueous humour and plasma in patients with POAG were reported [29]. A relation between homocysteine and IOP or NTG was not found [31,32]. For PEX glaucoma results are slightly different. A meta-analysis revealed that patients with PEX have a high prevalence of hyperhomocysteinemia [33]. Decreased levels of vitamin B6, B9 and B12 were found to be associated with elevated homocysteine levels in patients with PEX glaucoma [34]. One study that compared the levels of vitamin B6, B9 and B12 reported higher plasma levels of vitamin B6 in NTG and POAG patients, but no significant difference in serum levels of vitamin B9 and B12 between the groups [35]. The latter finding was in line with earlier studies [19,27]. Studies on dietary intake of vitamin B-complex and glaucoma did not reveal univocal results. For vitamin B1 the Rotterdam Study reported a protective effect on OAG, though the Study on osteoporotic fractures did not find any association between vitamin B1 and OAG [1,22,23]. The latter study also reported an association of vitamin B2 with OAG (OR [95% CI]: 0.39 [0.17–0.86]). For the association of dietary intake of vitamin B3, B6 or B9 with OAG no association was found [22,23]. Vitamin B9 intake may lower the risk of PEX probably by reducing homocysteine levels [36]. Concerning dietary intake of vitamin B12 none of the retrieved studies reported an association with OAG. One study reported no association of vitamin B12 intake with PEX glaucoma [36]. An interesting finding is that patients with vitamin B12 deficiency have a thinner retinal nerve fiber layer compared to controls. This thinning was correlated with plasma vitamin B12 levels [37].

Vitamin C is an essential nutrient involved in the repair of tissue. Four studies reported on the association of blood levels of vitamin C and glaucoma. In patients with NTG lower serum levels of vitamin C compared to controls have been reported [19], but for OAG results are conflicting [21,25,38]. In aqueous humour one study measured higher levels of vitamin C in OAG compared to controls [39], but two studies reported the opposite [40,41]. Oral vitamin C may increase its concentration in the aqueous humour [42]. Concerning the dietary intake of vitamin C results on the risk of glaucoma are contradicting. Two studies found a significant protective effect of vitamin C on OAG [21,22]. However, others did not find an association between vitamin C and POAG [1,23,24]. An important source of vitamin C is green leafy vegetables. Several studies provided evidence for an association of these foods with glaucoma (OR [95% CI]: 0.82 [0.69–0.97]; P-trend = 0.02) [43,44]. This is in line with the protective association with glaucoma for green collards and leaf cabbage found in two other studies (OR [95% CI]: 0.31 [0.11–0.91] and 0.43 [0.21–0.85], respectively) [22,23].

Vitamin D is important for certain minerals (e.g., Calcium). Five studies reported on the association of blood levels of vitamin D and glaucoma. In one study patients with glaucoma were reported to have lower serum 25-hydroxyvitamin D levels (OR [95% CI]: 0.89 [0.80–0.99]) and higher prevalence of vitamin D deficiency (OR [95% CI]: 2.09 [1.06–4.12]) compared to controls [45]. This is in agreement with the Korean National Health and Nutrition Examination Survey (KNHANES). They found a vertically reversed J-shaped association of serum 25-hydroxyvitamin D with OAG (OR [95% CI]: 1.61 [1.09–2.38] for the lowest quintile compared to the second highest quintile; P-trend [quadratic] = 0.02), with a more pronounced effect in males [46]. Similarly, two other studies showed lower serum 25-hydroxyvitamin D levels in POAG patients [47,48]. In contrast, the retrospective cross-sectional Kangbuk Samsung health study did not find any difference in total vitamin D levels between patients with and without glaucoma [49]. Also a nested case-control study revealed no associations of 25-hydroxyvitamin D levels with IOP [50]. Vitamin D-related metabolites and polymorphisms of the vitamin D receptor gene have been negatively associated with POAG [47,51]. Regarding the dietary intake of vitamin D results on the risk of glaucoma are less promising. Only few studies assessed this relation. The Study on osteoporotic fractures in African-Americans found no association of vitamin D with glaucoma (OR [95% CI]: 0.91 [0.45–1.83]) [22].

Vitamin E is seen as an important antioxidant. Six studies reported on the association of blood levels of vitamin E and glaucoma. One reported lower plasma levels of vitamin E in the POAG group [38]. One study reported no association [21], and two other studies found increased serum vitamin E levels in glaucoma patients [17,18]. For NTG, one study showed lower plasma levels of vitamin E in NTG patients [20], while other studies found no difference in plasma levels of vitamin E between patients with NTG and controls [19,21,52]. Concerning aqueous humour, lower levels of vitamin E have been reported in patients with POAG and PACG [41]. Studies on the dietary intake of vitamin E and its association with OAG revealed no significant associations [1,21,22,23,24].

The directions of the effect size between results of blood levels of vitamins on glaucoma, and of dietary intake of vitamins on glaucoma are not always in agreement with each other (Supplementary Tables S4 and S5 for OAG). This either suggests a poor association of vitamins with glaucoma or a poor correlation between blood levels of vitamins and intake of vitamins. In case of the former a meta-regression analysis would be helpful, however, due to low number of studies we did not perform an analysis as such. In case of the latter, a meta-analysis would be meaningful.

**Figure 2:**
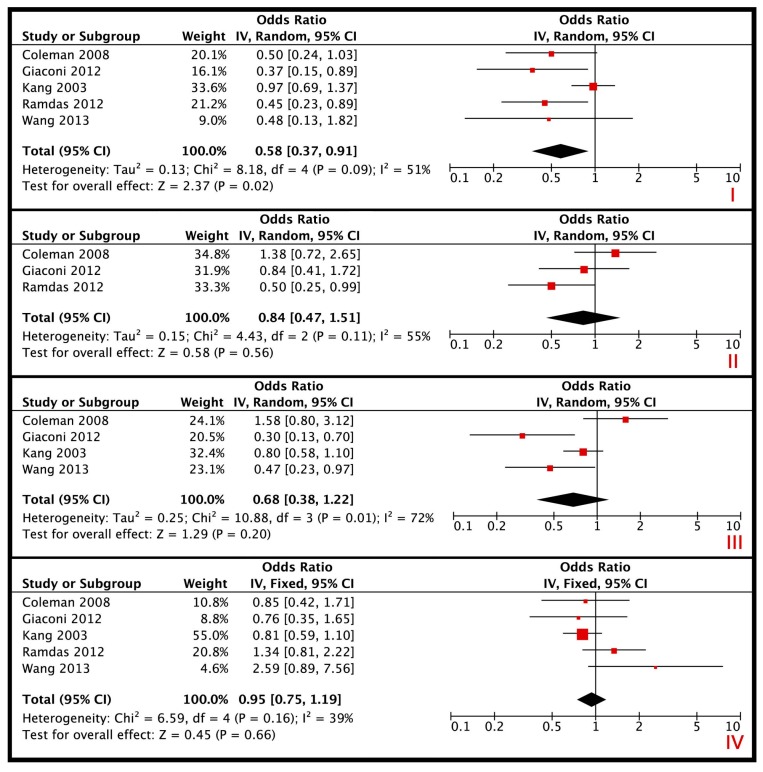
Meta-analyses for the association of vitamin A (I), B1 (II), C (III), and E (IV) with open-angle glaucoma. Black diamonds indicate the overall OR. The size of the red box is inversely proportional to the variance. Horizontal lines indicate 95% CI. The dashed vertical line in each panel shows the value for no effect (OR = 1.0).

Figure 2 displays the results of the meta-analysis. As only two studies reported an effect size for vitamin B2 and B3, and only one study reported an effect size for vitamin D on glaucoma, no meta-analysis was performed for these vitamins. A total of five eligible studies were included in the meta-analysis that assessed the dietary intake of vitamins and its association with OAG (Table 2). The prevalence of OAG in the study populations were in the range of 0.41–13.18% totaling 940 OAG cases and 123,697 controls. Therefore, the ORs of the studies could be interpreted as risk ratios. Dietary intake of vitamin A had a pooled OR [95% CI] of 0.58 [0.37–0.91] on OAG. However, the heterogeneity was high (I^2^ = 51%). After omitting the study that contributed to the heterogeneity, the pooled OR [95% CI] was 0.45 [0.30–0.68]; I^2^ = 0%). For vitamin B1, C and E no significant association with OAG was found (OR [95% CI]: 0.84 [0.47–1.51]; 0.68 [0.38–1.22]; 0.95 [0.75–1.19]; respectively). However, the meta-analysis for vitamin C showed high heterogeneity (I^2^ = 72%). After omitting the studies that significantly contributed to the heterogeneity, the pooled OR [95% confidence interval] was 0.39 [0.23–0.67] with no heterogeneity (I^2^ = 0%). Regarding green leafy vegetables a source for vitamin A, C and nitrate, a joint OR [95% CI] of 0.55 [0.30–1.02] was found with high heterogeneity (I^2^ = 67%). Again the study that contributed to the high heterogeneity was the Nurses’ Health Study and Health Professionals Follow-up Study [44]. Deleting this study from the random-effects model resulted in a joint OR [95% CI] of 0.39 [0.22–0.70] with no heterogeneity (I^2^ = 0%). Although this effect was based on only two studies, all three studies showed a significant protective association with OAG.

If we combine the studies on the association of blood levels of vitamins with OAG, and the studies on the association of dietary intake of vitamins with OAG, results are not very different. Supplementary Table S6 shows the level of evidence from the literature for the association of blood levels of vitamins and vitamin intake with OAG (following Supplementary Table S5). Of all vitamins vitamin A and C probably have a protective effect on OAG, and vitamin B1 is possibly associated with OAG.

## 4. Discussion

This review summarizes findings in the literature concerning the association of vitamins with glaucoma. Most of the studies focused on POAG. An important finding was the probable protective effect of vitamin A intake on OAG. The association of vitamin C with OAG did not reach statistical significance in the current meta-analysis unless heterogeneity was taken into account. Furthermore, dark green leafy vegetables, which contain vitamin A, C and nitrate, seem to have a beneficial effect in glaucoma. However, it is possible that nitrate is responsible for this effect. No consistent association was found for plasma or serum levels of any of the vitamins with glaucoma (Supplementary Tables S3 and S4). When combining studies on blood levels of vitamins with studies on dietary intake of vitamins, we report a probable association of vitamin A and C with OAG, and a possible association of vitamin B1 with OAG.

Several studies have implicated vitamins in affecting the course of common eye diseases. Vitamin A, B9, C, and E are well-known antioxidants and may prevent age-related eye disorders such as cataract and age-related macular degeneration [53,54]. It has been reported that administration of nicotinamide, a form of vitamin B3 (Table 1), has a strong neuroprotective effect in glaucoma independent of IOP [55]. Although studies on the dietary intake of vitamin B3 did not find an association with OAG [22,23], it should be noted that vitamins may indirectly influence other important parameters for OAG. For example, vitamin E has vasoregulatory effects via protein kinase C [56], which is, together with Rho/ROCK system activation, believed to be the main factor inducing aqueous humour outflow by trabecular meshwork cell relaxation in OAG [57]. According to the current literature it is likely that oxidative stress also plays a role in the pathogenesis of glaucoma. Systemic oxidative stress is associated with decreased ocular blood flow [58]. This association is related to the vascular permeability and results in a release of endothelin and nitric oxide (both oxidants). Blood levels of endothelin-1 have found to be significantly higher in patients with POAG [20]. The association of nitric oxide with glaucoma has been reported before [59]. Thus the nitrate-nitrite-nitric oxide pathway may play an important role in POAG. Nitrate is a source of nitric oxide and available in dark green leafy vegetables. In addition to nitrate, dark green leafy vegetables are also rich in vitamin A, C, and K. The present review suggested a beneficial association of vitamin A and C with OAG, but studies on vitamin K vs. OAG are currently lacking. Thus dark green leafy vegetables contain several relevant nutrients for OAG. This is emphasized by the fact that all retrieved studies on the association of green leafy vegetables with OAG found a significant association [22,23,43,44]. Another benefit patients with glaucoma may have from vitamin A is that vitamin A protects the conjunctiva and relieves dry eye symptoms caused by long-term application of IOP-lowering eye drops [60].

At present, there is no strong evidence to support a hypothesis that blood levels of vitamins impact glaucoma. An explanation could be the correlation between blood levels of vitamins and intake of vitamins, which depends on many co-factors. To mention a few: incorrect reported intake of vitamins from dietary supplements or food additives, different food processing techniques which may affect vitamin contents (e.g., exposure to high temperatures may lead to break down of some vitamins), various lifestyle factors (e.g., smoking, exercise, chronic low-grade inflammation) which may reduce vitamin levels, biases in the assessment of dietary vitamin intake, or potential influence of genetic variability on metabolism or absorption of vitamins [61]. For example, the *SLC23A2* gene, which plays a role in the absorption and accumulation of vitamin C in many tissues, has been associated with lower plasma concentrations of vitamin C and with higher risk of POAG [62]. If the correlation between blood levels of vitamins and dietary intake of vitamins is strong then the presented level of evidence of a possible association of vitamins with glaucoma would be poor. However, a meta-analysis showed that plasma vitamin C levels and dietary vitamin C intake are only modestly correlated (*r* = 0.4) [63]. Moreover, subjective judgment of the association of vitamins with OAG was not very different from the objective meta-analyses when taking studies on blood levels of vitamins into account. It should be noted that the results of studies on blood levels of vitamins are different between the several forms of glaucoma (POAG, PACG, NTG, and PEX glaucoma). The assessment of both blood levels and dietary intake is not standardized, which may contribute to the lack of correlation between blood levels and dietary intake. Especially the evaluation of vitamin D in this respect is difficult due to its endogenous synthesis, which could be why few studies have looked at vitamin D.

The quality of the studies was relatively high, but some concerns could be raised. The way patients were diagnosed with OAG differed between the included studies in the meta-analysis, and therefore some diagnostic bias might be present. Most studies based their OAG definition on a combination of optic nerve head abnormalities and glaucomatous visual field loss, but two studies classified their cases according to self-reports (Table 2). The self-reports were verified by checking the medical records and to further minimize misclassification participants had to have eye examinations in the past 2 years [24]. However, as mentioned in the Introduction, from population-based studies we know that half of the glaucoma cases are undiagnosed [64]. Furthermore, the prevalence of glaucoma was only 0.41% (Table 2), which is far lower than expected in their age group. This may have lead to an underestimation of their effect size. The sample size of their study resulted in a high weight in the current meta-analyses. Taken together this may have induced the heterogeneity, though omitting this study from the meta-analysis of vitamin A did not affect the results significantly. Cross-sectional studies are susceptible to bias because one cannot make clear determinations of cause and effect and often in secondary analyses of meta-analyses, those are separated analyzed, however, omitting these studies did not affect the results (data not shown).

Another drawback may be the different analyses for the nutrients. Nutrients were analyzed as tertiles [1], quartiles [21,22,23], or quintiles [24]. Therefore in the current study only the result of the highest intake was compared to the lowest intake of that nutrient, which served as the reference in all studies. Wang, et al. assessed the intake of supplements of vitamin A [21]. The amount of intake of their first quartile was comparable with the highest intake of the other studies. Including the results of this first quartile (OR [95% CI]: 0.55 [0.20–1.49] instead of 0.48 [0.13–1.82]) did not alter the results of the meta-analysis of vitamin A (data not shown).

Another limitation might be publication bias. To address this issue we performed a search in the abstract itinerary/meeting archives of the two largest congresses in ophthalmology: the Association for Research in Vision and Ophthalmology (ARVO) and the American Academy of Ophthalmology (AAO) annual meetings. The same search term of the current study was used and titles of abstract were screened from the meetings from 2010 to 2016. None of the retrieved abstracts showed contradictive results, however most studies focused on blood levels of vitamins (data not shown).

In conclusion, although there is sufficient evidence that oxidative stress plays an important role in the pathogenesis of different types of glaucoma, studies on the blood levels of vitamins in patients with glaucoma are inconclusive and do not correlate with results on the effect of dietary intake of vitamins on glaucoma. Moreover, studies on the association of the dietary intake of vitamins with glaucoma are scarce. However, vitamin A intake probably has a protective effect on glaucoma. Vitamin C may have a comparable effect on glaucoma as well. This review highlighted new targets and strategies for future research aiming to assess the influence of nutrition on glaucoma. Especially, randomized clinical trials are required to verify the role of these vitamins in glaucoma and to investigate whether nutritional supplements can become part of the treatment of glaucoma. Although a nutritional supplement for glaucoma might be an additional option to treat persons with advanced glaucoma, a clear effect on the disease as seen in age-related macular degeneration probably does not exist.

## Figures and Tables

**Table 1:** Overview of investigated vitamins retrieved from the systematic review.

Vitamin	Vitamer Chemical Name	Remarks	Relevance to the Eye	Main Sources	Rich Food Sources
A	Retinol (A1)	Preformed retinols (or active form)—is immediately available to the body	Important for rhodopsin found in the retina for visual phototransduction, and protects against free radicals i.e., acts as an antioxidant	Animal products	Liver, eggs, cheese, butter, dark green vegetables (spinach) and carrots
		Precursors (or provitamins)—has to be metabolized into an active form	Important for rhodopsin found in the retina for visual phototransduction, and protects against free radicals i.e., acts as an antioxidant	Plant products	Vegetables and fruits with orange pigments (known as carotenoids)
B-complex	Thiamin (B1)	Some flavonoids may antagonize vitamin B1	Serve as coenzymes in catabolism of amino acids, carboxylase, cell division and growth, and DNA synthesis and repair in every cell of the body	Animal/plant products	Yeast extract, cereal grains (especially in the outer layer of the grain and in the germ), meat (pork), nuts, peas, and beans
	Riboflavin (B2)	Primary coenzyme form of vitamin B6	Used in the treatment of corneal ectasia for strengthening of corneal collagen tissue. It can also be used as a prophylaxis for migraine: a potential risk factor for glaucoma	Animal/plant products	Milk, cheese, eggs, green leafy vegetables, almonds, and mushrooms
	Niacin (B3)	May be converted to nicotinamide, which is another form of vitamin B3	Involved in vasodilatation of among others retinal arterioles. It improves endothelial dysfunction	Animal/plant products	Fish, dates, nuts, and cereal grains
	Pyridoxin (B6)	Dietary vitamin B6 cannot be used without vitamin B2 (Riboflavin)	Involved in neurotransmitter synthesis. Plays a role in the treatment of gyrate atrophy of the choroid and retina	Animal/plant products	Meat (pork), bananas, chickpeas, potatoes, and pistachios
	Folate (B9)	Synthetic form, used in supplements and food processing, is called Folic acid	When combined with vitamin B12 it reduces homocysteine levels, which induces vascular injury, alterations in extracellular matrix remodeling, and neuronal cell death	Plant products	Cereal grains (especially in the outer layer of the grain and in the germ), dark green leafy vegetables, nuts, peas, and beans
	Cobalamin (B12)	Should be in balance with folic acid and iron	Coenzyme involved in the metabolism of every cell of the body. Deficiency can results in elevated homocysteine levels, optic neuropathy, and irreversible damage to nervous system	Animal products	Fish, red meat, eggs, and cheese
C	Ascorbic acid	Oxidized form of vitamin C is reduced by glutathione, which helps maintain vitamin C in a reduced (active) form	Enzymatic cofactor for collagen synthesis and very effective in scavenging of reactive oxygen species, i.e., vitamin C is an important non-enzymatic antioxidant	Plant products	Peppers, green leafy vegetables, and in many fruits (kiwis, strawberries, oranges, guavas, and papaya)
D	Cholecalciferol	Most important subtype in humans is vitamin D3. In the liver vitamin D is converted to 25-hydroxyvitamin D, which is used as a biomarker	Responsible for intestinal absorption of several minerals including calcium, iron, magnesium, and zinc	Sunlight exposure	Mushrooms, cod liver oil, fish, and cereals
E	Tocopherol	Excessive vitamin E may lead to vitamin K * deficiency resulting in bleedings	Plays a role in the oxidation of low-density lipoproteins (LDL) and prevents the production of damaging free radicals. Therefore vitamin E is seen as an important antioxidant. Deficiency might result in peripheral neuropathy and retinopathy	Plant products	Nuts (especially almonds), sunflower oil and seeds, avocados, and dark green leafy vegetables

* = None of the retrieved studies assessed the relation between vitamin K and glaucoma.

**Table 2:** Characteristics of the included studies for the meta-analyses.

Study	Study Design		Age (Years)	Race	N (Patients)			Prevalence (%)	Definition of Glaucoma	Adjusted Covariates
					Glaucoma (Undefined)	OAG	N (Total)	Cases/Total		
Coleman 2008 [23]	cross-sectional	cohort	>65	Caucasian/African	95		1155	8.23	Based on ONH photographs and VF	Study sites, age, race and ethnicity, education, smoking, alcohol, walking for exercise, BMI, self-rated health status, self-reported diabetes, self-reported hypertension, and age-related macular degeneration.
Giaconi 2012 [22]	cross-sectional	case-control	>65	African	77		584	13.18	Based on ONH photographs and VF	Study sites, age, education, smoking, alcohol, walking for exercise, BMI, self-rated health status, self-reported diabetes, and self-reported hypertension.
Kang 2003 [24]	prospective	cohort	>40	Caucasian/African		474	116,484	0.41	Self-reported glaucoma, confirmed with medical records	African heritage, diabetes, hypertension, BMI, physical activity, alcohol intake, and smoking.
Ramdas 2012 [1]	prospective	cohort	≥55	Caucasian		91	3502	2.60	Based on slitlamp, ONH (photographs and scans), and VF	Age, gender, IOP, IOP-lowering treatment, BMI, beta-carotene, vitamin B1, B12, E, and magnesium.
Wang 2013 [21]	cross-sectional	cohort	≥40	Multiracial	203		2912	6.97	Self-reported glaucoma	Age, demographic variables, smoking, alcohol, self-reported general health condition, and self-reported diabetic retinopathy.

OAG = open-angle glaucoma; VF = Visual field; ONH = Optic nerve head appearance; IOP = Intraocular pressure; BMI = Body mass index.

## Supplementary Materials

The following are available online at http://www.mdpi.com/2072-6643/10/3/359/s1, Table S1: Summary of the retrieved studies from the systematic review; Table S2: Quality assessment of included studies according to the Newcastle-Ottawa Scale *; Table S3: Blood levels of studies on vitamin blood levels and glaucoma; Table S4: Overview of retrieved studies on the association of vitamin blood levels with open-angle glaucoma; Table S5: Overview of retrieved studies on the association of vitamin blood levels (in bold) and intake with open-angle glaucoma; Table S6: Evidence for association of vitamins with open-angle glaucoma: Judgement of investigators.
