# Supplementary material for: The Effect of Vitamins on Glaucoma: A Systematic Review and Meta-Analysis
Source: Nutrients. 2018 Mar 16;10(3):359. doi: 10.3390/nu10030359 (PMC5872777; doi:10.3390/nu10030359)
Supplement: Supplementary file 1 [file nutrients-10-00359-s001.pdf]

Supplementary appendix

The effect of vitamins on glaucoma: a systematic review and meta-analysis

Ramdas WD, et al.

Contents:

SUPPLEMENTARY TABLE 1: Summary of the retrieved studies from the systematic review

SUPPLEMENTARY TABLE 2: Quality assessment of included studies according to the Newcastle-Ottawa Scale*

SUPPLEMENTARY TABLE 3: Blood levels of studies on vitamin blood levels and glaucoma

SUPPLEMENTARY TABLE 4: Overview of retrieved studies on the association of vitamin blood levels with open-angle glaucoma

SUPPLEMENTARY TABLE 5: Overview of retrieved studies on the association of vitamin blood levels (in bold) and intake with open-angle glaucoma

SUPPLEMENTARY TABLE 6: Evidence for association of vitamins with open-angle glaucoma: Judgement of investigators

SUPPLEMENTARY TABLE 1. Summary of the retrieved studies from the systematic review

STUDY	STUDY DESIGN		N (patients, not eyes!)							PREVALENCE	DEFINITION OF GLAUCOMA	METHOD USED FOR EXPOSURE VARIABLE
			Glaucoma (undefined)	(P)OAG	NTG	PACG	PEX/PEG	controls	TOTAL	cases/total		
Arar, et al. 2016	cross-sectional	case-control		20				20	40	50.00	Based on VF, ONH, slitlamp	Electrochemiluminescence
Asregadoo, et al. 1979	cross-sectional	case-control		38				12	50	0.76	*No full text available*	*No full text available*
Belmonte, et al. 2011	cross-sectional	case-control		48	13			78	139	43.88	Clinical diagnosis	HPLC, CLEIA
Burgess, et al. 2015	cross-sectional	case-control		72				72	144	50.00	Based on VF, ONH, slitlamp	Ultra-high resolution mass spectrometry with C18 liquid chromatography
Coban, et al. 2014	cross-sectional	case-control	77					40	117	65.81	Based on VF, ONH, slitlamp	CLEIA and food questionnaires
Coleman, et al. 2008	cross-sectional	cohort	95					1060	1155	8.23	Based on VF, ONH	Block Food Frequency Questionnaire
Cumurcu, et al. 2006	cross-sectional	case-control		25	18		24	19	86	77.91	Based on IOP, VF, ONH, slitlamp	CLEIA
Engin, et al. 2010	cross-sectional	case-control	160					31	191	83.77	(multicenter study on clinical parameters)	HPLC
Giaconi, et al. 2012.	cross-sectional	case-control	77					507	584	13.18	Two glaucomaspecialist based on VF, ONH	Block Food Frequency Questionnaire
Goncalves, et al. 2015	cross-sectional	case-control		150				164	314	47.77	Based on IOP, VF, ONH	Radioimmunoassay
Goyal, et al. 2014*	cross-sectional	case-control		30		30		30	90	66.67	Clinical diagnosis; age-matched cataract controls	Spectrophotometry
Gye, et al. 2016	cross-sectional	cohort	2431					161598	164029	1.48	Fundusphotographs with ISGEO criteria	*Laboratory analysis not specified*
Iqbal, et al. 1999*	cross-sectional	case-control							58	NA	Clinical diagnosis; patients undergoing cataractsurgery	HPLC coupled ECD
Kang, et al. 2003	prospective	cohort		474				116010	116484	0.41	self-reported glaucoma confirmed with medical records	Semiquantitative Food Frequency Questionnaire
Kang, et al. 2014	prospective	cohort					399	119802	120201	0.33	self-reported glaucoma confirmed with medical records	Semiquantitative Food Frequency Questionnaire
Kang, et al. 2016	prospective	cohort		1483				103504	104987	1.41	self-reported glaucoma confirmed with medical records	Semiquantitative Food Frequency Questionnaire

Krefting, et al. 2014	RCT	case-control; population-based							129	NA	*NA; IOP-measurements in low vs. high blood levels*	Liquid chromatography double mass spectrometry
Lee, et al. 1977*	cross-sectional	case-control		35				4	39	89.74	Samples during glaucoma surgery	Colorimetric method, HPLC
Leibovitzh, et al. 2016	cross-sectional	cohort; population-based	42					11808	11850	0.35	Clinical diagnosis	Fluorescence polarization immunoassay technology
Li, et al. 2016	Review and meta-analysis		NA	NA	NA	NA	NA	NA	NA	NA	NA	NA
Lopez-Riquelme, et al. 2014	cross-sectional	case-control		48	15			75	138	45.65	Clinical diagnosis	CLEIA, HPLC, ELISA
Lv, et al. 2016	cross-sectional	case-control		71				73	144	49.31	Clinical diagnosis	ELISA
Marcus, et al. 2011	Review and meta-analysis		NA	NA	NA	NA	NA	NA	NA	NA	NA	NA
Moise, et al. 2012	cross-sectional	cohort	25					475	500	5.00	Based on IOP, VF, ONH	Semiquantitative Food Frequency Questionnaire
Ramdas, et al. 2012	prospective	cohort; population-based		91				3411	3502	2.60	Based on VF, ONH, slitlamp	Semiquantitative Food Frequency Questionnaire
Ren, et al. 2006	cross-sectional	case-control		10				8	18	55.56	Clinical diagnosis; controls were healthy siblings	Spectrophotometry, HPLC
Roedl(1), et al. 2007*	cross-sectional	case-control		39				39	78	50.00	Clinical diagnosis; age-matched controls	HPLC, Immunoassay
Roedl(2), et al. 2007	cross-sectional	case-control					70	70	140	50.00	Clinical diagnosis; matched controls	HPLC, Immunoassay
Rossler, et al. 2010	cross-sectional	case-control			42			42	84	50.00	Clinical diagnosis; age-matched controls	HPLC, Immunoassay
Turgut, et al. 2010	cross-sectional	case-control		34	48		34	40	156	74.36	Based on IOP, VF, ONH, slitlamp	HPLC, CLEIA
Turkyilmaz, et al. 2013	cross-sectional	case-control							90	NA	*NA; RNFL-thickness in low vs. high bloodlevels*	CLEIA
Wang, et al. 2013	cross-sectional	cohort	203					2709	2912	6.97	self-reported glaucoma	HPLC
Xu, et al. 2011	Review and meta-analysis		NA	NA	NA	NA	NA	NA	NA	NA	NA	NA
Xu, et al. 2012	Review and		NA	NA	NA	NA	NA	NA	NA	NA	NA	NA

	meta-analysis											
Yoo, et al. 2014	cross-sectional	cohort	410 glaucoma suspects	290				5394	5684	5.10	Based on IOP, VF, ONH, slitlamp; with ISGEO criteria	Gamma counter with radioimmunoassays
Yuki, et al. 2010	cross-sectional	case-control			47			44	104	57.69	Clinical diagnosis	HPLC coupled ECD
Zanon-Moreno, et al. 2013	cross-sectional	case-control		250				250	500	50.00	Based on IOP, VF, ONH, slitlamp	ECD

* = These studies only analyzed aqueous humour levels; RCT = Randomized controlled trial; POAG = Primary open-angle glaucoma; NTG = Normal-tension glaucoma; PACG = Primary angle-closure glaucoma; PEX/PEG = Pseudoexfoliation/PEX-glaucoma; VF = Visual field; ONH = Optic nerve head; IOP = Intraocular pressure; RNFL = Retinal nerve fiber layer; HPLC = High performance liquid chromatography; CLEIA = Chemiluminescence enzyme immunoassay; ECD = Electrochemical detection; ELISA = Enzyme-linked immunosorbent assay

SUPPLEMENTARY TABLE 2. Quality assessment of included studies according to the Newcastle-Ottawa Scale*

Study	Design	Selection	Comparability	Outcome (cohort) / Exposure (case-control)	Total score (max. 9)
Arar, et al. 2016	Case-control	1	0	3	4
Belmonte, et al. 2011	Case-control	2	0	3	5
Burgess, et al. 2015	Case-control	3	2	3	8
Coban, et al. 2014	Case-control	3	1	3	7
Coleman, et al. 2008	Cohort	4	1	3	8
Cumurcu, et al. 2006	Case-control	2	2	3	7
Engin, et al. 2010	Case-control	2	1	3	6
Giaconi, et al. 2012.	Cohort	3	2	3	8
Goncalves, et al. 2015	Case-control	3	2	3	8
Goyal, et al. 2014	Case-control	2	1	3	6
Gye, et al. 2016	Cohort	4	1	2	7
Iqbal, et al. 1999	Case-control	1	0	2	3
Kang, et al. 2003	Cohort	3	1	2	6
Kang, et al. 2014	Cohort	3	1	2	6
Kang, et al. 2016	Cohort	3	2	2	7
Krefting, et al. 2014	RCT	4	2	3	9
Lee, et al. 1977	Case-control	0	0	3	3
Leibovitzh, et al. 2016	Cohort	3	2	3	8
Li, et al. 2016	Review	NA	NA	NA	NA
Lopez-Riquelme, et al. 2014	Case-control	3	1	3	7
Lv, et al. 2016	Case-control	4	1	3	8
Marcus, et al. 2011	Review	NA	NA	NA	NA
Moise, et al. 2012	Cohort	3	0	2	5
Ramdas, et al. 2012	Cohort	4	1	3	8
Ren, et al. 2006	Case-control	2	0	3	5
Roedl(1), et al. 2007	Case-control	3	1	3	7
Roedl(2), et al. 2007	Case-control	3	2	3	8
Rossler, et al. 2010	Case-control	3	2	3	8
Turgut, et al. 2010	Case-control	4	1	3	8
Turkyilmaz, et al. 2013	Case-control	4	1	3	8
Wang, et al. 2013	Cohort	2	2	3	7
Xu, et al. 2011	Review	NA	NA	NA	NA
Xu, et al. 2012	Review	NA	NA	NA	NA
Yoo, et al. 2014	Cohort	4	2	3	9
Yuki, et al. 2010	Case-control	3	1	3	7
Zanon-Moreno, et al. 2013	Case-control	3	2	3	8

* = For each of the sections Selection, Comparability, and Outcome/Exposure, a maximum of 4, 2, and 3 points could be given, respectively. Thus the maximum score was 9. A higher score means higher quality.

SUPPLEMENTARY TABLE 3. Blood levels of studies on vitamin blood levels and glaucoma. Presented as mean±standard deviation unless stated otherwise.

Study^reference no.	Vit.	Glaucoma undefined	POAG	NTG	PEX/PEG	Controls	Unit	P-values / Remarks	Significant (p<0.05)
Belmonte 2011[17]	A		37.7±13.2	30.4±15.8		37.9±12.0	ug/dL	p=0.061	No
Lopez-Riquelme 2014[20]	A		39.7±13.3	30.4±15.8		37.9±12.0	ug/dL	p=0.061	No
Yuki 2010[19]	A			82.1±26.7		77.1±30.1	ug/dL	p=0.41	No
Asregadoo 1979[25]	B1		X			X	NA	Only p-value mentioned (p<0.001)	Yes
Roedl(1) 2007[29]	B6		12.64±6.50			13.46±9.00	ng/mL	p=0.65	No
Roedl(2) 2007[34]	B6				10.29±5.73	12.54±6.40	ng/mL	p=0.029	Yes
Turgut 2010[35]	B6		30.22±12.15	30.50±11.29	22.81±11.71	20.09±5.54	ug/mL	POAG: p=0.025; NTG: p=0,01; PEX: p>0.05	Yes/No
Belmonte 2011[17]	B9		6.7±3.6	5.9±3.4		5.7±2.8	pg/mL	p=0.173	No
Coban 2015*[26]	B9	7.86±3.15				6.9±3.1	ng/mL	p=0.144	No
Cumurcu 2006[27]	B9		6.24±2.88	5.97±1.85	4.26±1.69	5.93±1.70	ng/mL	POAG/NTG: p>0.05; PEX: p<0.009	No/Yes
Lopez-Riquelme 2014[20]	B9		6.8±3.7	5.9±3.4		5.7±2.8	ng/mL	p=0.173	No
Roedl(1) 2007[29]	B9		11.76±4.38			12.07±5.78	ng/mL	p=0.79	No
Roedl(2) 2007[34]	B9				8.59±4.27	11.51±4.74	ng/mL	p<0.001	Yes
Turgut 2010[35]	B9		11.59±9.40	9.71±3.86	10.82±4.92	9.00±6.39	ng/mL	p>0.05	No
Yuki 2010[19]	B9			8.7±4.3		8.0±3.1	ng/mL	p=0.37	No
Belmonte 2011[17]	B12		404.3±198.2	471.7±177.6		425.7±137.7	pg/mL	p=0.385	No
Coban 2015*[26]	B12	231±135.19				272.5±84.54	pg/mL	p=0.016	Yes
Cumurcu 2006[27]	B12		232.84±67.55	262.33±85.94	209.37±104.44	261.84±126.22	pg/mL	POAG/NTG/PEX: p>0.05	No
Lopez-Riquelme 2014[20]	B12		404.2±198.2	471.7±177.6		425.7±137.7	pg/mL	p=0.708	No
Roedl(1) 2007[29]	B12		461.7±228.9			478.7±266.3	pg/mL	p=0.76	No
Roedl(2) 2007[34]	B12				323±129	413±170	pg/mL	p=0.001	Yes
Turgut 2010[35]	B12		368.24±262.65	344.46±247.84	277.16±139.08	345.55±201.75	pg/mL	p>0.05	No
Asregadoo 1979[25]	C		X			X	NA	Only p-value mentioned (p>0.05)	No
Yuki 2010[19]	C			4.6±4.0		6.3±3.9	ug/mL	p=0.04	Yes
Zanon-Moreno 2013[38]	C		10.0±1.6			12.0±1.7	ug/mL	Gene-interaction (p<0.001 for blood level)	
Arar 2016[48]	D		32.31			64.17	nmol/L	p<0.05	Yes
Goncalves 2015[45]	D		42.9±25.7			49.4±29.5	nmol/L	p=0.039	Yes
Gye 2016[49]	D	16.59±7.01				15.85±6.96	ng/mL	p<0.001	Yes
Lv 2016[47]	D		26.37±5.83			30.43±3.91	ng/mL	p<0.001	Yes
Yoo 2014[46]	D	X	X			X	ng/dL	Quintiles	NA
Belmonte 2011[17]	E		1050.4±330.5	739.9±288.0		1008.7±232.5	ug/dL	p=0.001	Yes
Lopez-Riquelme 2014[20]	E		1050.4±330.6	739.9±288.0		1008.7±232.5	ug/dL	p=0.001	Yes
Ren 2006[52]	E		25.3±7.4			28.5±7.8	umol/L	No p-value mentioned	NA
Yuki 2010[19]	E			1.5±0.6		1.5±0.6	mg/dL	p=0.83	No
Zanon-Moreno 2013[38]	E		10.7±1.7			11.4±1.8	ug/mL	Gene-interaction (p<0.001 for blood level)	Yes
Engin 2010[18]	A, E	X				X	NA	Associated with clinical parameters	NA
Wang 2013[21]	A, C, E	X				X	ug/dL	Quintiles	NA

X = No absolute value applicable; * = median±standard deviation; Vit. = Vitamin; POAG = Primary open-angle glaucoma; NTG = Normal-tension glaucoma; PEX/PEG = Pseudoexfoliation/PEX-glaucoma

SUPPLEMENTARY TABLE 4. Overview of retrieved studies on the association of vitamin blood levels with open-angle glaucoma

Studies	Lower levels	No effect	Higher levels
Vitamin	significant	non-significant	significant
A		Belmonte 2011<br>Engin 2010<br>Lopez-Riquelme 2014<br>Wang 2013<br>Yuki 2010	
B1	Asregadoo 1979		
B2			
B3			
B6	Roedl(2) 2007	Roedl(1) 2007	Turgut 2010
B9	Roedl(2) 2007	Belmonte 2011<br>Coban 2015<br>Cumurcu 2006<br>Lopez-Riquelme 2014<br>Roedl(1) 2007<br>Turgut 2010<br>Yuki 2010	
B12	Coban 2015<br>Roedl(2) 2007	Belmonte 2011<br>Cumurcu 2006<br>Lopez-Riquelme 2014<br>Roedl(1) 2007<br>Turgut 2010	
C	Yuki 2010<br>Zanon-Moreno 2013	Asregadoo 1979<br>Wang 2013	
D	Arar 2016<br>Goncalves 2015<br>Lv 2016<br>Yoo 2014*	Gye 2016	Yoo 2014*
E	Zanon-Moreno 2013	Ren 2006<br>Yuki 2010<br>Wang 2013	Belmonte 2011<br>Engin 2010<br>Lopez-Riquelme 2014

* = J-shaped association;
Roedl(2) 2007: focused on pseudoexfoliation glaucoma
Yuki 2010: focused on normal-tension glaucoma

SUPPLEMENTARY TABLE 5. Overview of retrieved studies (except of meta-analyses studies) on the association of vitamin blood levels (in bold) and intake with open-angle glaucoma, presented as first author with year of publication [sample size (N); POAG prevalence (%)] quality-score.

Studies	Lower risk		No effect (OR or RR close to 1.00)	Higher risk	
Vit.	significant	non-significant		non-significant	significant
A	Giaconi 2012 [584; 13.18] 8<br>Ramdas 2012 [3502; 2.60] 8	Coleman 2008 [1155; 8.23] 8<br>Wang 2013 [2912; 6.97] 7	Kang 2003 [116484; 0.41] 6<br>**Belmonte 2011 [139; 43.88] 5**<br>**Lopez-Riquelme 2014 [138; 45.65] 7**<br>**Wang 2013 [2912; 6.97] 7**		
B1	Ramdas 2012 [3502; 2.60] 8<br>**Asregadoo 1979 [50, 79.00] NA**	Giaconi 2012 [584; 13.18] 8		Coleman 2008 [1155; 8.23] 8	
B2	Coleman 2008 [1155; 8.23] 8	Giaconi 2012 [584; 13.18] 8			
B3		Giaconi 2012 [584; 13.18] 8		Coleman 2008 [1155; 8.23] 8	
B6		Giaconi 2012 [584; 13.18] 8<br>**Roedl(1) 2007 [78; 50.00] 7**	Coleman 2008 [1155; 8.23] 8		**Turgut 2010 [156; 74.36] 8**
B9	Giaconi 2012 [584; 13.18] 8	**Roedl(1) 2007 [78; 50.00] 7**<br>**Cumurcu 2006 [86; 77.91] 7**	Coleman 2008 [1155; 8.23] 8	**Belmonte 2011 [139; 43.88] 5**<br>**Coban 2014 [117; 65.81] 7**<br>**Lopez-Riquelme 2014 [138; 45.65] 7**<br>**Turgut 2010 [156; 74.36] 8**	
B12	**Coban 2014 [117; 65.81] 7**	**Belmonte 2011 [139; 43.88] 5**<br>**Lopez-Riquelme 2014 [138; 45.65] 7**<br>**Roedl(1) 2007 [78; 50.00] 7**	Ramdas 2012 [3502; 2.60] 8	**Cumurcu 2006 [86; 77.91] 7**<br>**Turgut 2010 [156; 74.36] 8**	
C	Giaconi 2012 [584; 13.18] 8<br>Wang 2013 [2912; 6.97] 7<br>Moise 2012 [500; 5.00] 5<br>**Zanon-Moreno 2013 [500; 50.00] 8**	Kang 2003 [116484; 0.41] 6	**Wang 2013 [2912; 6.97] 7**<br>**Asregadoo 1979 [50, 79.00] NA**	Coleman 2008 [1155; 8.23] 8	
D	**Arar 2016 [40; 50.00] 4**<br>**Goncalves 2015 [314; 47.77] 8**<br>**Lv 2016 [144; 49.31] 8**<br>**Yoo 2014* [5684; 5.10] 9**		Giaconi 2012 [584; 13.18] 8<br>**Gye 2016 [164029; 1.48] 7**		**Yoo 2014* [5684; 5.10] 9**
E	**Zanon-Moreno 2013 [500; 50.00] 8**	Coleman 2008 [1155; 8.23] 8<br>Giaconi 2012 [584; 13.18] 8<br>Kang 2003 [116484; 0.41] 6<br>**Ren 2006 [18; 55.56] 5**	**Wang 2013 [2912; 6.97] 7**	Ramdas 2012 [3502; 2.60] 8<br>Wang 2013 [2912; 6.97] 7	**Belmonte 2011 [139; 43.88] 5**<br>**Lopez-Riquelme 2014 [138; 45.65] 7**

Vit. = Vitamin; OR = Odds ratio; RR = Risk ratio; * = J-shaped association;

SUPPLEMENTARY TABLE 6. Evidence for association of vitamins with open-angle glaucoma: Judgement of investigators

Vitamin	WDR	JSS	CAW	Mean score	Association with OAG
A	Definite	Possible	Probable	2.000	Probably associated
B1	Probable	Not	Possible	1.000	Possibly associated
B2	Possible	Possible	Possible	1.000	Possibly associated
B3	Not	Not	Not	0.000	Not associated
B6	Not	Not	Possible	0.333	Not associated
B9	Possible	Not	Possible	0.667	Possibly associated
B12	Not	Not	Possible	0.333	Not associated
C	Probable	Possible	Probable	1.667	Probably associated
D	Possible	Possible	Probable	1.333	Possibly associated
E	Not	Not	Not	0.000	Not associated

OAG = Open-angle glaucoma
